# Cases of Traumatic Erysipelas, with Constitutional Disturbance of Various Degrees

**Published:** 1826-11

**Authors:** 

**Affiliations:** St. Thomas's Hospital.


### TRAUMATIC ERYSIPELAS.

*Cases of Traumatic Erysipelas, with Constitutional Disturbance of various degrees, successfully treated by* Mr. Travers, at St. Thomas's Hospital.

Case I. (*Symptoms moderate.*) *Erysipelatous Inflammation, and Chain of Abscesses, of the Leg and Thigh, following a lacerated Wound of the Toes.*

June 27th, 1826.—Dagleish Forster, ætatis twenty-one, a muscular man, of healthy appearance, was admitted into St. Thomas's Hospital, with a wound, one inch and a half long, at the root of the second and third toes of the right foot, inflicted by two deals which had fallen upon them. Adhesive plaster was applied, and the wound was granulating.

July 6th.—Was this morning attacked with pain in the head, sickness, and a shivering fit followed by a hot paroxysm. An erysipelatous redness appeared at the instep and shin, which in the course of the day extended rapidly up the leg. He complains of great pain in the head. The bowels have been very costive for several days past.

To take the house-physic, and apply a poultice to the wound.

8th.—The redness is still extending; the sickness and fever continue.

℞. Mist. Potass. Citrat. cum Liq. Ant. Tart.

10th.—The erysipelatous redness extends up the leg and thigh, principally on the inner side, following the course of the absorbents; there are two or three enlarged and inflamed glands at the inside of the knee and in the groin; the leg and thigh are swollen, tender, and painful; he complains of much pain in the head, and deafness; he has sickness, pain in the abdomen and tenderness on pressure, particularly about the epigastric region; the pulse is 120 in the minute, full and soft; the tongue is loaded with a thick yellow crust, the skin hot and dry; his sleep is much disturbed; great thirst. The bowels have been much relaxed by the medicine.

℞. Mist. Camph. ℥jss.; Liq. Ammon. Acet. ʒss. M. sextâ quâque horâ sumend.—℞. P. Opii gr. j. omni nocte.

12th.—Rather better. The erysipelatous inflammation has not extended, and the pain in the head has abated. There yet remains considerable tenderness of the abdomen; the bowels again confined.

Hyd. Subm. p. et statim.—Haust. Sennæ post quater horas.—℞. Antim. Tartar. gr. j.; Aquæ distil. ℥ iv. M. sumatur coch. j. amplum secunda quâque horâ.

15th.—Inflammation has subsided. An abscess has formed at the calf, where fluctuation is obscurely perceived: on puncturing it, a quantity of pus, with shreds of dead cellular membrane, escaped. There remains great swelling of the limb. General health improved; no pain; tongue cleaner and moist; pulse frequent, small and soft; spirits much depressed.

℞. Dec. Cinchon. ℥jss.; Acid. Nitric. dil. m. v. M. ter die sumendus.

19th.—A second abscess, which had formed at the inside of the leg, was this day punctured. A circumscribed blush of inflammation appears upon the inside of the thigh.

22d.—Two more abscesses opened at the inside of the leg. His spirits improved; sleeps well; has a good appetite.

℞. Extr. Cinchon. ℈j.; Dec. Cinchon. ℥ x.; Tinct. Ejusd. Co. ℨj. M. ter die sumend.—Mutton-chop and porter daily.

27th.—The wound of the toes is foul, with slight surrounding inflammation.

Applicetur Catap. cum Lotion Acid. Nitric dil.

—An abscess opened at the inside of the thigh, and another at the inner ankle.

August 5th.—The abscesses are all quite healed: health and strength daily increasing.

September 13th.—Progressively improved from the date of last report. A small portion of the first phalanx of the third toe exfoliated. Wound healed; health entirely re-established.

Case II. (*Symptoms severe.*) *Inflammation of the Arm and Forearm, with Suppuration and severe Disturbance of the Constitution, following Contusion of the Elbow.*

June 1st, 1826.—Thomas Stephens, hackney coachman, ætatis forty-five, admitted with a large abscess of the left arm and forearm, extending above and below the elbow-joint. The patient is stout and muscular, accustomed to the free use of porter and spirits, but has good health. On the 26th May, while in the act of mounting his coach-box, he fell backwards, and struck his elbow against the pavement. This was followed by great pain and inflammation of the arm, but he continued to follow his occupation till the 29th, when the pain became so severe that he was compelled to desist. He then applied a bran-poultice, and kept his bed till his admission.

Fluctuation very evident on the radial side of the forearm, which is much swollen, and of a regular and intense redness. The inflammation extends to the middle of the arm. A free opening was made through the integuments and fascia, allowing the escape of a great quantity of pus, with shreds of sloughy cellular membrane.

Apply a roller lightly above and below the opening.—Catap. Lini c. Fol. Papav.—Sumatur M. Sennæ C. pro re nata.

June 2d.—Tongue slightly furred; pulse quick. Does not complain of any uneasiness except in the arm.

3d.—The discharge of pus from the wound copious and healthy. A sinus extends above the elbow, communicating with the wound in the forearm. Tongue still furred, but no other constitutional symptoms; bowels open.

5th.—Last evening he was delirious: complained of great pain in the head and in the inflamed parts, which were much swollen. Was continually getting in and out of bed, but laid quiet when spoken to, and was at intervals rational. Countenance pallid and anxious; respiration hurried; pulse slow, soft and feeble; skin cool and moist; bowels open. Ordered by the apothecary, Mist. Potass. Citrat. cum Liq. Antim. Tart.—This morning the symptoms, as above described, continue. An incision has been made in the arm above the outer condyle.

℞. Ammon. Carb. gr. v.; Dec. Cinchon. ℥jss.; R. Ejusd. ʒij.; Tr. Opii, m. v. M. sextis horis sumend.—Omit the Saline Mixture.

6th.—Slight delirium; no pain in the head; feels very faint and weak; pulse seventy, full and soft; tongue yellowish white; slight thirst; sleeps very little.

7th.—Pulse eighty, greater fulness and strength; tongue moist, white centre, clean edges; bowels confined; has slept several hours. Has less wandering, and his countenance is tolerably composed; there is also less irritation about the arm.

℞. Ol. Ricini ʒvj. statim.—Cerev. Oj. per diem.—Rep^r alia.

8th.—Continued improvement. Copious and healthy discharge from the arm.

10th.—Very weak. The redness and swelling of the arm have quite subsided; the discharge is less, and the wound is rapidly healing.

28th.—Left the hospital in tolerable health and strength, the arm being quite sound, but very weak.

Case III. (*Symptoms very severe.*) *Erysipelatous Inflammation of the Right Upper Extremity and Side, following a Fracture of the Metacarpal Bone of the Right Thumb.*

December 4th, 1824.—Jeremiah Duggin, ætatis sixty-three, an Irish labourer, living very freely and irregularly, was admitted into St. Thomas's Hospital, with a fracture through the middle of the metacarpal bone of the right thumb, produced by falling on his hands from a scaffold. Considerable swelling and tension followed the injury, but the displacement of the fractured portions and the crepitus were distinct. Twenty leeches were applied immediately, and afterwards a poultice with Goulard's wash; and he was ordered Pil. Coloc. cum Cal. gr. v. h. s. and house-physic in the morning. The swelling gradually subsided.

8th.—Two small splints were applied to the thumb, and bound on with a roller.

10th.—A slight blush of inflammation about the right elbow-joint. Patient's health appears to sympathise.

12th.—Swelling and redness much increased; pain very great; pulse 100, hard and sharp; tongue yellow; great thirst; skin hot and dry; bowels confined.

Remove the splints, and apply simply Cerat. Sapon.—℞. Pulv. Scammon. cum Calom. gr. xv. statim sum.—Mist. Sennæ C. cras mane.—Hirudines xij. brachio admovend.—Catap. cum Lot. alb. (Liq. Plumbi Acet. dil.)

15th.—The swelling and redness have continued to spread both above and below the elbow. The whole arm and forearm, and a portion of the pectoral region, are now inflamed; the redness more intense in some parts than others, forming patches, with paler surrounding portions, so that the integuments have a variegated appearance. On pressure with the finger an indentation is made, which remains for some minutes. Above the joint where the inflammation first appeared, an obscure fluctuation is perceptible. Pulse 120, small and sharp; tongue very foul; thirst considerable. The bowels, which have been with difficulty acted upon ever since his admission, now require repeated doses of M. Sennæ C. before any effect can be produced. He complains of great pain in the arm, is very restless, and can get no sleep.

℞. Infus. Rosæ ℥j.; Magn. Sulph. ʒjss. bis die sum.—P. Ipec. C. gr. x. omni nocte sum.—Applicetur brachio Catap. cum Fotû Papav.

16th.—Fluctuation more evident in front of the elbow-joint, where an incision an inch long was made, from which serum only escaped.

17th.—A considerable quantity of serum has flowed from the wound; the surrounding inflammation has in some degree abated. The redness and boggy swelling has extended from the pectoral region as far down the side as the crista ilii. The man now appears sinking: the utmost anxiety is depicted on his countenance; the eyes hollow, the nose pinched; respiration difficult and hurried; the lips parched; tongue dry, and covered with a thick brown crust; pulse fluttering, and indeed scarcely perceptible; there is also a slight degree of comatose delirium.

18th.—Symptoms as above: redness of the side less intense. An incision, three inches long, through the integuments of the forearm, from the elbow downwards, exposed the fascia, which was sloughy, of a dull yellow colour: a small quantity of pus, and much serum, escaped.

℞. Sp. Æther. Nitr. ʒj. cum Mist. Camph, ℥j. t. d. sum.—P. Opii gr. j. o. n.—Vin. rubri ℥vj. indies.—Omit the former medicines.

20th.—The discharge altered from serum to healthy pus, and in tolerable quantity. Has passed a better night, and is on the whole improved. Redness of side subsiding.

23d.—The wound at the bend of the elbow dilated, and the fascia at the side of the forearm divided, when a small quantity of pus escaped, and portions of dead cellular membrane. Has complained of slight sickness.

24th.—The sickness continues: dislikes his wine, which he refuses to take. Substitute for it brandy ℥iv. in the day.

25th.—A considerable quantity of dark-coloured blood escaped from the wound in the forearm,—perhaps a pint. The whole fascia and integuments of the forearm are elevated from the muscles, and distended by a highly fetid gas, and a sanious fluid mixed with coagula. The man has passed a sleepless night, and has vomited frequently: his general appearance is very unfavourable, and, from his having become weaker and more emaciated, he is thought to be even worse than on the 17th instant. The opening in the forearm was dilated from the elbow to the wrist, when a considerable quantity of sanies escaped: the wound was then covered with simple cerate, and a roller applied to the arm.

℞. Haust. Salin. Efferv. cum Tinct. Opii m. x. sextis horis sumend.

26th.—General appearance rather improved. Has passed a better night; sickness less frequent; healthy pus is discharged from the wound; the surface of the exposed muscles is florid. Portions of sloughy fascia and cellular membrane, which were hanging loose, have been gently drawn out and cut off: the swelling of the upper arm subsiding. He has no appetite, but drinks freely of milk.

Habeat Aquæ Vitæ ℥ vj. per diem.—Quinæ Sulph. gr. iv. ter quotidie.

From this time the improvement was progressive and rapid: the wound was covered with simple dressings, and the arm and forearm carefully rolled. Till January 1st, the discharge was very great, requiring fresh dressings twice daily: it gradually diminished, and on the 3d the integuments of the forearm adhered throughout; the edges of the wound, however, had retracted nearly two inches apart, exposing the extensor muscles of the hand: the exposed surface assumed a very beautiful appearance, small vessels carrying red blood shooting across from one side to the other, with at first apparently nothing to support them, but on the following day covered with a thin layer of semi-transparent lymph. On the upper arm, the integuments did not adhere so rapidly, in consequence of a collection of pus at the posterior edge of the axilla, the outlet for which was down the arm, through a canal beneath the fascia. This collection was opened on the 5th, allowing the escape of a quantity of very offensive pus and sloughs. The discharge then became more healthy, and continued for a few days, when the edges adhered.

February 6th.—The wound of the forearm has completely and firmly cicatrised: it is still covered with simple dressings, and rolled. The wound at the outer margin of the deltoid muscle, which had been healed more than three weeks, has become inflamed, and a blush of inflammation extends down the arm, accompanied by a thickened and indurated state of the integuments. On examination, a small opening was found in the cicatrix, through which a probe passed one inch and a half down the arm : this sinus was dilated, and in a few days healed. By this his recovery was retarded, though for a short time only.

On the 17th March he was discharged, the arm being quite sound, and his health good.

